# Prognostic Assessment of COVID-19 in the Intensive Care Unit by Machine Learning Methods: Model Development and Validation

**DOI:** 10.2196/23128

**Published:** 2020-11-11

**Authors:** Pan Pan, Yichao Li, Yongjiu Xiao, Bingchao Han, Longxiang Su, Mingliang Su, Yansheng Li, Siqi Zhang, Dapeng Jiang, Xia Chen, Fuquan Zhou, Ling Ma, Pengtao Bao, Lixin Xie

**Affiliations:** 1 Chinese PLA General Hospital Medical School Of Chinese PLA College of Pulmonary and Critical Care Medicine Beijing China; 2 DHC Mediway Technology Co Ltd Beijing China; 3 The 940th Hospital of Jiont Logistics Support Force of Chinese People's Liberation Army Lanzhou China; 4 The 980th Hospital of Jiont Logistics Support Force of Chinese People's Liberation Army Shijiazhuang China; 5 Peking Union Medical College Hospital Beijing China; 6 College of Pulmonary and Critical Care Medicine Chinese PLA General Hospital Beijing China

**Keywords:** COVID-19, ICU, machine learning, death prediction model, factor analysis, SHAP, LIME

## Abstract

**Background:**

Patients with COVID-19 in the intensive care unit (ICU) have a high mortality rate, and methods to assess patients’ prognosis early and administer precise treatment are of great significance.

**Objective:**

The aim of this study was to use machine learning to construct a model for the analysis of risk factors and prediction of mortality among ICU patients with COVID-19.

**Methods:**

In this study, 123 patients with COVID-19 in the ICU of Vulcan Hill Hospital were retrospectively selected from the database, and the data were randomly divided into a training data set (n=98) and test data set (n=25) with a 4:1 ratio. Significance tests, correlation analysis, and factor analysis were used to screen 100 potential risk factors individually. Conventional logistic regression methods and four machine learning algorithms were used to construct the risk prediction model for the prognosis of patients with COVID-19 in the ICU. The performance of these machine learning models was measured by the area under the receiver operating characteristic curve (AUC). Interpretation and evaluation of the risk prediction model were performed using calibration curves, SHapley Additive exPlanations (SHAP), Local Interpretable Model-Agnostic Explanations (LIME), etc, to ensure its stability and reliability. The outcome was based on the ICU deaths recorded from the database.

**Results:**

Layer-by-layer screening of 100 potential risk factors finally revealed 8 important risk factors that were included in the risk prediction model: lymphocyte percentage, prothrombin time, lactate dehydrogenase, total bilirubin, eosinophil percentage, creatinine, neutrophil percentage, and albumin level. Finally, an eXtreme Gradient Boosting (XGBoost) model established with the 8 important risk factors showed the best recognition ability in the training set of 5-fold cross validation (AUC=0.86) and the verification queue (AUC=0.92). The calibration curve showed that the risk predicted by the model was in good agreement with the actual risk. In addition, using the SHAP and LIME algorithms, feature interpretation and sample prediction interpretation algorithms of the XGBoost black box model were implemented. Additionally, the model was translated into a web-based risk calculator that is freely available for public usage.

**Conclusions:**

The 8-factor XGBoost model predicts risk of death in ICU patients with COVID-19 well; it initially demonstrates stability and can be used effectively to predict COVID-19 prognosis in ICU patients.

## Introduction

COVID-19 is a new and severe infectious disease that has spread to 34 provinces and cities in China and over 30 countries worldwide [1,2]. After the entire nation of China fought against COVID-19, by early May 2020, the numbers of patients with COVID-19 had greatly decreased in almost all provinces and cities in China. However, in mid-June, a new outbreak of COVID-19 cases occurred in Beijing, the capital of China. Government efforts have now brought the overall spread of COVID-19 under control. It is clear that COVID-19 is an infectious disease that requires ongoing attention from the medical community, governments, and the public to prevent future outbreaks. As of the end of June 2020, more than 10,000,000 COVID-19 cases had been recorded worldwide. Therefore, an evaluation and early warning system for COVID-19 prognosis is urgently needed, especially for critically ill patients.

COVID-19 cases are classified as mild, moderate, severe, or critical [3]. At present, most studies of COVID-19 have focused on risk factor analysis and mortality prediction for mild and moderate cases, which comprise a large proportion of patients with COVID-19 [4-8]. However, 14% to 20% of cases are severe or even critical [1,9], and the mortality rate of these patients is as high as 50% [10]. Few studies have reported risk factor prediction and mortality analysis for severe and critical patients with COVID-19. COVID-19 predictive models are rapidly entering the academic literature. These include predictive models that are mainly used to identify high-risk groups in the general population [11-13], diagnostic models that are used to detect COVID-19 [14-16], and models used to predict mortality, serious disease progression, etc [17-20]. The most common predictors of the diagnosis and prognosis of COVID-19 are age, body temperature, lymphocyte count, and lung imaging characteristics. The estimated C indices of these predictive models are between 0.65 and 0.99. Although the estimated C indices of some models appear to be ideal, all the models are rated as being at high risk of bias, mainly because of the high risk of model overfitting. Moreover, many of the report descriptions are vague. Most reports do not include a description of the study population or the intended use of the model, and very few evaluations of the calibration of model predictions were made [21].

The theoretical core of machine learning analysis is the data mining algorithm. Various data mining algorithms based on different data types and formats can more scientifically represent the characteristics of the data and can better penetrate the data trends and recognized values [22]. On this basis, one of the most important application areas is predictive analysis, which involves identifying features (in machine learning, “features” refers to individual characteristics of the data) from mechanical learning, establishing models through science, and then running new data through the models to predict future data [23]. In this study, we clarify that the established model is used to predict the prognosis of patients with COVID-19 in the intensive care unit (ICU). The model must be continuously optimized and evaluated. In terms of evaluation, we checked the accuracy and calibration of the model. Moreover, to improve the interpretability of the black box model, we also used SHapley Additive exPlanations (SHAP) and Local Interpretable Model-Agnostic Explanations (LIME) to explain the prediction model; therefore, the prediction model not only predicts prognostic outcomes but also gives a reasonable explanation for the prediction, which can greatly enhance users’ trust of the model.

## Methods

### Study Design and Data Source

Vulcan Hill Hospital, located in Wuhan, Hubei Province, is a special hospital that was built by the Chinese government to treat patients with COVID-19. Construction on the hospital started on January 24, 2020, and was completed on February 1; the hospital entered use on February 2, and it officially closed on April 15. During this period, a total of 3063 patients with laboratory-confirmed hospitalized cases of COVID-19 were admitted. For this study, data of 3063 patients with COVID-19 admitted to Vulcan Hill Hospital were extracted from hospital medical records and screened for eligibility. The study extracted 100 relevant variables, such as baseline patient information, clinical diagnosis, vital signs, laboratory test results, medical advice, and nursing care, as candidate variables for predictors [24]. We established a study cohort of 123 critically ill patients admitted to the ICU, and 2940 patients who did not enter the ICU were excluded. Considering the problem of predictors and the timing of the outcome measurement, we used the time the patient entered the ICU to calculate the first test value of all candidate predictors upon entering the ICU. The output of our study is the prognostic outcome of these critically ill patients, and this outcome is based on the ICU death record in the electronic medical record. After further checking the admission records, we included data of 123 critically ill patients admitted to the ICU, including 65 (52.8%) who survived and 58 (47.2%) who died. We randomly used 80% of these data as the training set, and the remaining 20% were used as the validation set. For the training set data, we completed the statistics and proper processing of missing values, the identification and processing of noise data, and the standardization of all predictive variables. The validation set was processed in exactly the same way as the training set. Then, we researched and analyzed the feature selection, model training, model evaluation, and model interpretation.

It is worth noting that this study adhered to the TRIPOD (Transparent Reporting of a multivariable prediction model for Individual Prognosis Or Diagnosis) statement for reporting, and completion of the model construction and verification was guided by PROBAST (Prediction model Risk Of Bias ASsessment Tool) [25,26]. This study was approved by the ethics committee of Vulcan Hill Hospital; the requirement for informed consent was waived.

### Predictor Variables and Data Preprocessing

**Figure 1:**
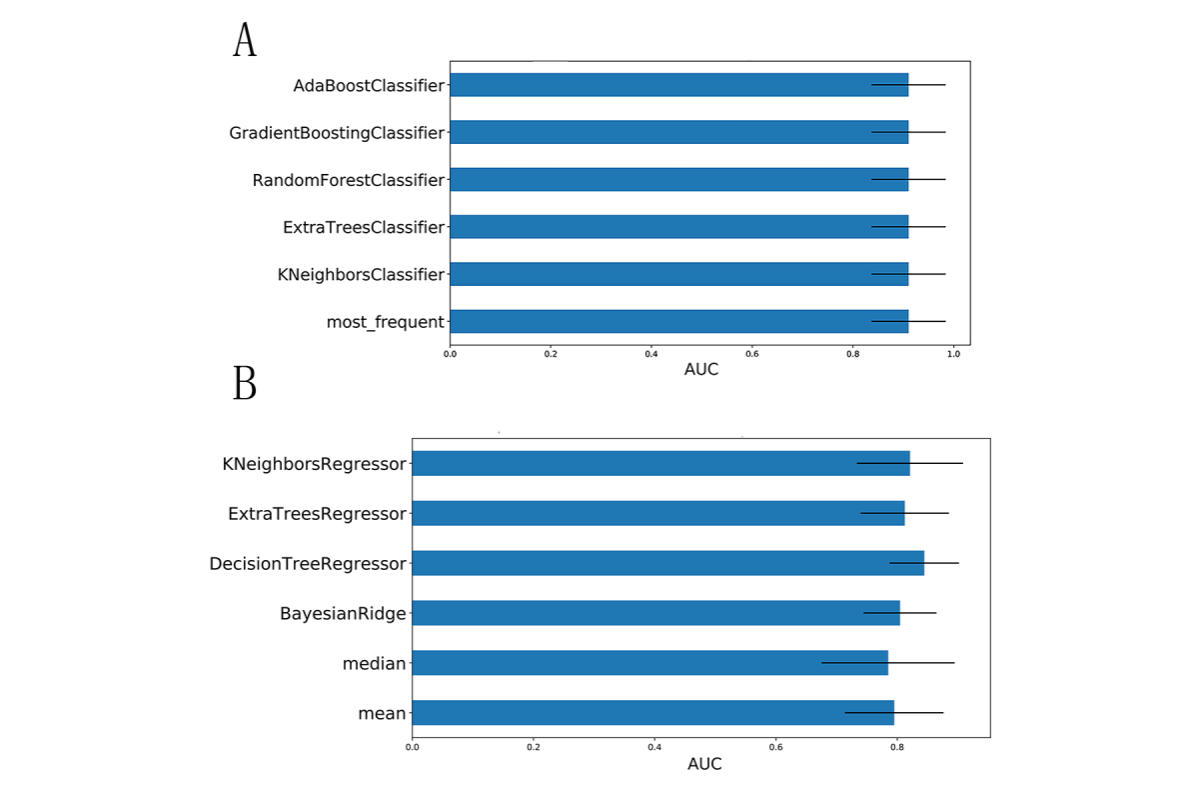
(A) Comparison of the AUC values obtained after using logistic regression as a carrier and filling in discrete features with multiple missing filling methods. (B) Comparison of the AUC values obtained after continuous feature filling by multiple missing filling methods using logistic regression as a carrier. AUC: area under the receiver operating characteristic curve.

In this study, a total of 100 candidate predictive features were collected, and the test results were the first measured value after the patient entered the ICU. Among these features, 5 features with a missing ratio greater than 30% were excluded, and the remaining features were filled with missing values using appropriate methods. Because missing data may lead to loss of useful information and even create instability of the model realization, it is more difficult to analyze the model results with missing data; therefore, we carried out a cautious missing value interpolation strategy. We used the Iterative Imputer tool developed by scikit-learn to perform multiple imputations for missing values. The Iterative Imputer uses an algorithm to model each missing value feature as a function of other features. It uses the predicted value of the function as an estimate. In each step, one feature is selected as the output y, and all other features are selected as the input X. Then, a regressor is trained on X and y to predict the missing value of y. The area under the receiver operating characteristic curve (AUC) values corresponding to each padding method were found to be basically the same. We also applied the K Neighbors Regressor, Decision Tree Regressor, Bayesian Ridge, and Extra Trees Regressor regression algorithms as predictors to complete missing value filling. Moreover, we attempted mean filling and median filling and fed the above six imputation results into the traditional logistic regression model to calculate and compare the areas under the receiving operator characteristic curve (AUC) of their respective prediction results. The results in Figure 1 show that the best filling method is multiple imputation, with Decision Tree Regressor as the regression method; therefore, this method was finally used to fill in the missing data for the continuous features. The missing data are provided in Multimedia Appendix 1. In addition, we drew box plots for continuous features and used IQR criteria to filter and replace outliers. Finally, to obtain more reliable prediction performance, the continuous data were standardized by the z score standardization method.

### Feature Selection and Statistical Analysis

The categorical variables of the queue data were expressed as n (%). Continuous variables that satisfy normal distribution were expressed as mean (SD); otherwise, medians and quartiles were used. All characteristics were evaluated statistically, and two-sided differences in *P* values <.05 were considered statistically significant. Differences between categorical variables were compared using the chi-square test or Fisher exact test as needed. The independent sample *t* test was used to compare continuous variables that satisfy normal distribution, while the Wilcoxon test was used for nonnormally distributed continuous variables. Statistically significant features were selected for further correlation analysis. For redundant features with strong correlation, factor analysis was used to confirm the collinearity of the variables, classify the collinearity as a latent factor, and then calculate the eigenvalues, visualize the gravel map of the eigenvalues, and select the feature root. Values >1 and the first few principal components where the slope decreases were used as principal component factors to eliminate redundant features and to find more efficient, concise, and precise feature combinations, thereby improving the generalization and practical capabilities of the model, as described previously [27]. Note that factor analysis requires the data to be suitable according to the Bartlett sphere test and Kaiser-Meyer-Olkin test.

### Derivation and Validation of the Models

A conventional logistic regression method and four popular machine learning classification algorithms, including adaptive boosting (AdaBoost), gradient boosting decision tree (GBDT), eXtreme Gradient Boosting (XGBoost), and CatBoost, were applied in the present study to model the data. The model built by the algorithm uses constant parameter optimization and model evaluation to compare the fitting effects of each model and to select the best model as the risk prediction model. Model optimization is a method that combines grid search and five-fold cross-validation to visualize the AUC values of the model and the standard deviation with the parameters and selects the parameter values corresponding to the best AUC value as the model parameters [28].

A good model explanation must be presented for the black box model. This study is based on the SHAP algorithm, which calculates the marginal contribution of a feature when it is added to the model and then considers whether the factor is different in all factor sequences [29]. The marginal contribution fully explains the influence of all factors included in the model for model prediction and distinguishes the attributes of the factors (risk factors and protective factors).

Finally, validation queue data were used to evaluate the prediction performance of the model and calculate the AUC, threshold, Youden index, 95% CI, SD, and *P* value of the AUC, accuracy, sensitivity, specificity, positive predictive value (PPV), negative predictive value (NPV), positive likelihood ratio (PLR), and negative likelihood ratio (NLR) of the model for the test set. A calibration curve was drawn, the calibration degree of the model was measured, and the degree of consistency between the predicted risk and the actual risk of the model was evaluated. A good calibration degree indicates that the predicted value of the model is closer to the actual probability of the outcome, from the interpretation of the model to the prediction of random samples, as demonstrated previously [30]. The model, code, and parameters are provided in Multimedia Appendix 2.

### Interpretation of the Model for Prediction of Random Samples

Advanced machine learning models are usually “black boxes.” When the internal operations of a model are unknown, users do not trust the reliability of the model for making predictions. Although it is known that the accuracy of cross-validation of these models is very high, correlation is still sometimes found between the verification data and the training data due to improper methods, especially when there are few samples. Therefore, cross-validation is no longer the only indicator for evaluating trust. If the rationale by which a model predicts a single sample can be intuitively perceived, users can better trust or distrust single sample prediction. The LIME algorithm was implemented with this concern in mind. This linear model is used to locally approximate a black box model by giving weights to the disturbance input; thus, the observation model gives a basis for interpretation of the sample prediction results [31]. In the present study, we randomly sampled the test set and used the LIME algorithm to fit the predictive behavior of the model to the sample to verify the rationality of the basis of the model for predicting results.

## Results

### Study Population and Baseline Characteristics

**Figure 2:**
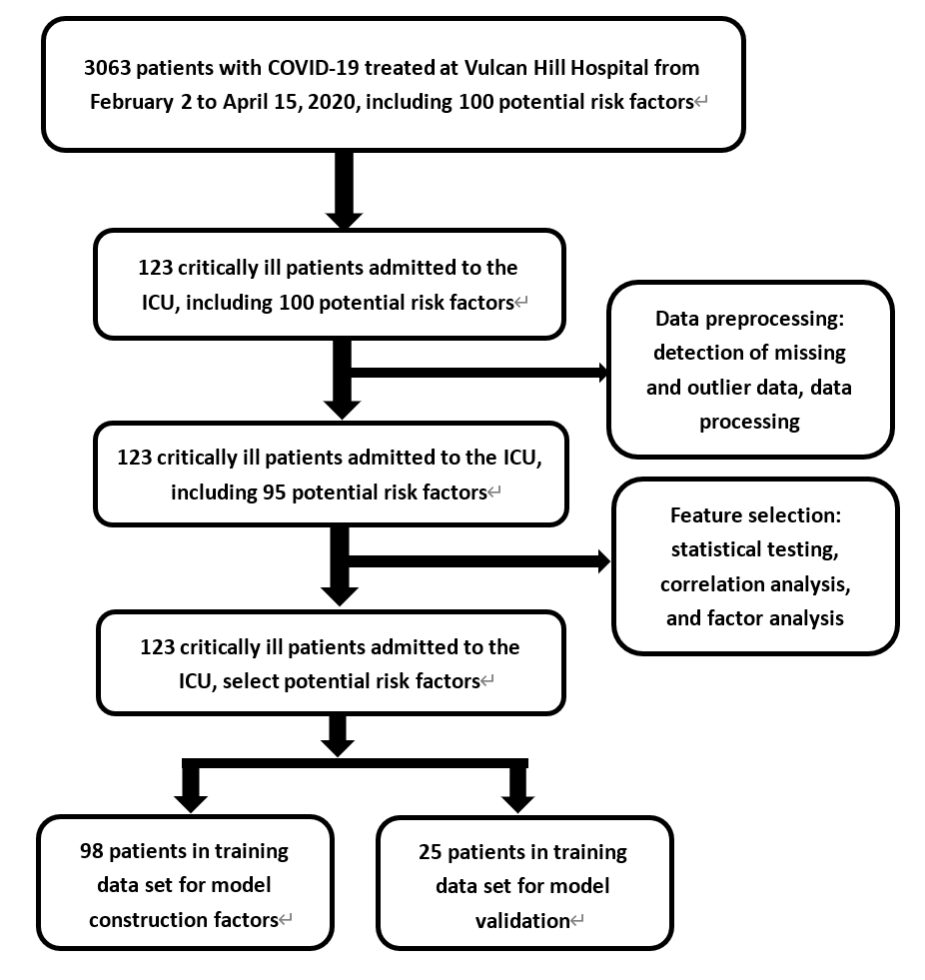
Flowchart of the data and feature selection.

**Figure 3:**
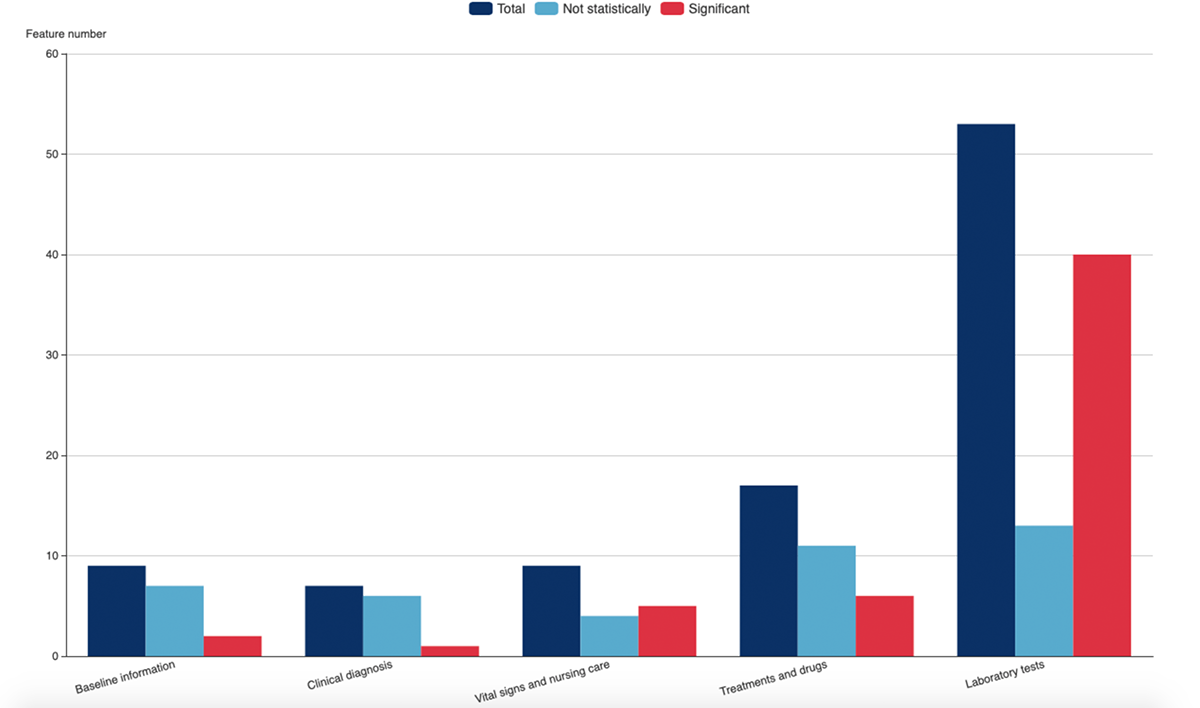
Statistical analysis of the screening results of all potential risk factors in the study cohort. The figure shows the screening of candidate variables in the five dimensions of patient baseline information, clinical diagnosis and vital signs, laboratory tests, medical advice, and nursing care.

Data from 3063 patients with COVID-19 treated at Vulcan Hill Hospital from February 2 to April 15, 2020, were analyzed retrospectively. A total of 69/3063 deaths occurred (2.3%). The final analytic sample included 123 critically ill patients admitted to the ICU, including 85 critically ill patients (69.1%), 65 surviving patients (52.8%), and 58 patients who died (47.2%). The outcome variable was determined as the prognostic outcome of critically ill patients, and the outcome was based on the death record in the electronic medical record. 100 related variables were established as candidate variables for predictors. Figure 2 shows a flowchart of the overall process of data and feature screening. Figure 3 and Multimedia Appendix 3 list the results comparing all potential risk factors in the study cohort. Overall, the mean age of the patients in the cohort was 69.8 years (SD 11.1), and 79/123 patients (64.2%) were male. Data analysis revealed significant differences between patients in 7 discrete factors, namely ventilator use, critical illness, vasoactive drugs, carbapenem use, antibiotic resistance, anti–gram-positive cocci, and hemodiafiltration; significant differences were found in 46 continuous factors.

### Predictor Selection

By selecting the abovementioned statistically significant factors for correlation analysis, the correlation coefficient matrix heat map (Multimedia Appendix 4) of the features shows that the top five features that were negatively correlated with the outcomes are prothrombin time percentage activity, blood oxygen saturation, lymphocyte percentage (LYM%), albumin level (ALB), and percentage of basophils (BASO%); the top five characteristics that were positively correlated with outcomes are lactate dehydrogenase, alpha-hydroxybutyrate dehydrogenase, C-reactive protein, neutrophil percentage (NEUT%), and original thrombin time. In addition, strong correlations were found between many features. For example, the correlation coefficient between the prothrombin time (PT) and international standardized ratio reached 0.999; therefore, it was necessary to reduce redundant features.

**Figure 4:**
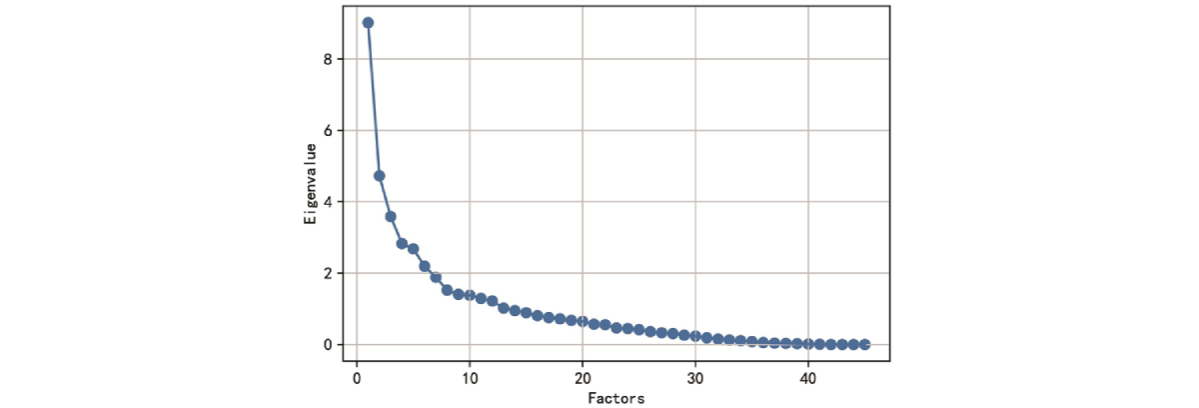
Distribution diagram of the correlations between the feature value and the number of features; when the feature value is >1 and the slope change becomes slow, the number of features is 8.

Factor analysis and visualization of the characteristic root gravel map and load matrix (Figure 4 and Multimedia Appendix 5) revealed that the eight principal component factors were the most predictive; for example, the correlation between the characteristic prothrombin time and the second main factor reached 0.97. Considering the convenience and practicability of using the prediction model, clinical experience and actual comparisons were combined to finally select eight features to represent the eight principal component factors, namely LYM%, PT, lactate dehydrogenase (LDH), total bilirubin (T-Bil) , eosinophil percentage (EOS%), creatinine (Cr), NEUT%, and ALB. The Kaiser-Meyer-Olkin test gave a value of 0.5714 and Bartlett's test of sphericity showed a significance level of *P*<.001, indicating that the factor analysis is effective.

### Machine Learning Algorithm Comparison and Best Model

**Figure 5:**
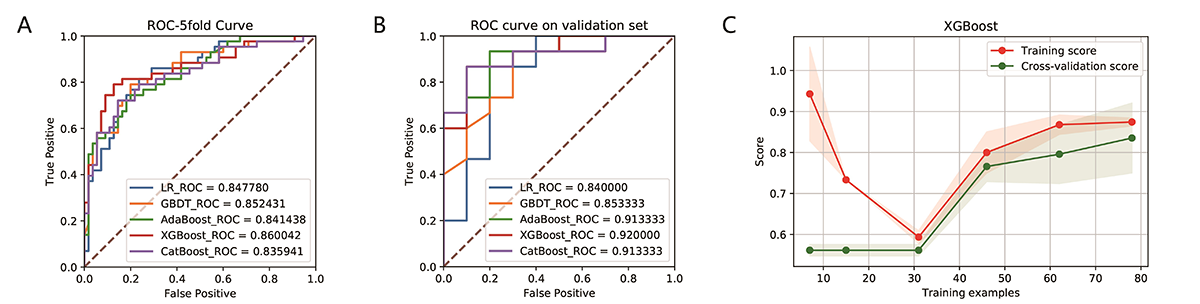
ROC curves showing the fitting performance (A) and prediction performance (B) of the LR, CatBoost, GBDT, XGBoost, and AdaBoost prediction models based on the eight important features in the training data set and the test data set. (C) The learning curve on the training set showing the learning process of the XGBoost model. The red line is the fitting effect of the model on the overall training set, and the green line is the fitting effect of the model on the training set with 5-fold cross-validation. The two curves finally merge near 0.85, indicating that the model is well fitted for training. AdaBoost: adaptive boosting; GBDT: gradient boosting decision tree; LR: logistic regression; ROC: receiver operating characteristic; XGBoost: eXtreme Gradient Boosting.

Comparing the AUCs of the logistic regression and four machine learning algorithms for 5-fold cross-validation on the training set (Figure 5), it can be found that the AUC values of each algorithm are similar; however, the AUC value of the XGBoost algorithm is higher. The XGBoost algorithm reflects a good learning curve on the training set, effectively preventing overfitting. In terms of prediction performance, the results of the logistic regression and four machine learning algorithms on the test set show AUCs of 0.92 for XGBoost, 0.9133 for CatBoost, 0.9133 for AdaBoost, 0.85 for GBDT, and 0.84 for LR; the best prediction performance was observed with XGBoost. In addition, the AUC, threshold, Youden index, 95% CI, SD, and *P* value of the AUC, accuracy, sensitivity, specificity, PPV, NPV, PLR, and NLR values of each model in the test data set are listed in Table 1. In summary, the results of the test data set show that the XGBoost model demonstrates the best performance based on eight salient features. The Youden index value of this model is 0.6667.

**Table 1:** Summary of prediction results of multiple models on the test set.

Value	Models				
	Logistic regression	AdaBoost^a^	GBDT^b^	XGBoost^c^	CatBoost
AUC^d^	0.84	0.9133	0.85	0.92	0.9133
Threshold	0.3962	0.4283	0.4583	0.4478	0.5063
Youden index	0.6667	0.7333	0.6333	0.7667	0.7667
95% CI of the AUC	0.6556-1.0	0.8024-1.0	0.6997-1.0	0.8142-1.0	0.7997-1.0
SD of the AUC	0.094	0.0566	0.0784	0.054	0.058
*P* value of the AUC	.003	<.001	.002	<.001	<.001
Accuracy	0.76	0.76	0.76	0.84	0.84
Specificity	0.8	0.9	0.8	0.9	0.8
Sensitivity	0.7333	0.6667	0.7333	0.8	0.8667
Positive predictive value	0.8462	0.9091	0.8462	0.9231	0.8667
Negative predictive value	0.6667	0.6429	0.6667	0.75	0.8
Positive likelihood ratio	3.6667	6.6667	3.6667	8	4.3333
Negative likelihood ratio	0.3333	0.3704	0.3333	0.2222	0.1667

^a^AdaBoost: adaptive boosting.

^b^GBDT: gradient boosting decision tree.

^c^XGBoost: eXtreme Gradient Boosting.

^d^AUC: area under the receiver operating characteristic curve.

### Model Validation and Predictor Parameters

**Figure 6:**
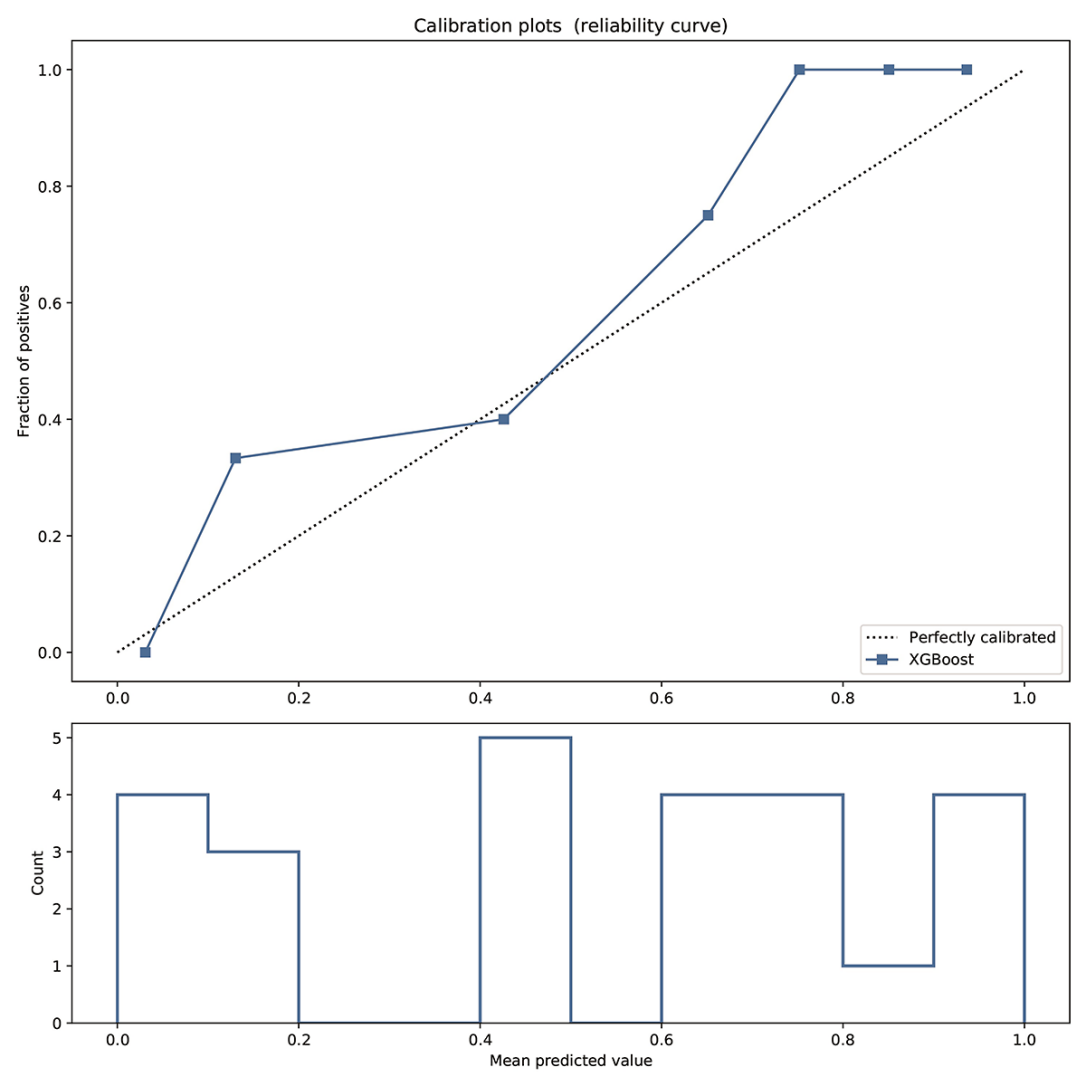
Calibration curve reflecting the degree of consistency between the predicted risk and the actual risk of the XGBoost model. The predicted curve of the model fits well with the diagonal, indicating that the predicted value of the model is basically close to the actual probability of the outcome.

The prediction behavior of XGBoost in the test set was visualized. The calibration curve (Figure 6) shows that the predicted risk of the XGBoost model is in good agreement with the actual risk. The predicted value of the model is close to the actual probability of the outcome. The details of the optimal model parameters constructed by the XGBoost algorithm can be viewed in Multimedia Appendix 2.

### Interpretation and Evaluation of the Machine Learning Model

**Figure 7:**
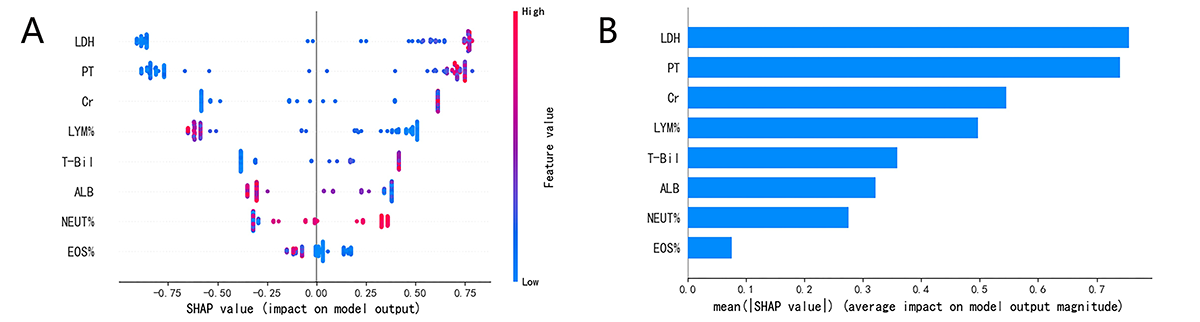
The XGBoost model based on the SHAP algorithm. (A) The attributes of the features in the black box model. Each line represents a feature, and the abscissa is the SHA*P* value, which represents the degree of influence on the outcome. Each dot represents a sample. The redder the color, the greater the value of the feature, and the bluer the color, the lower the value. (B) Ranking of feature importance indicated by SHAP. ALB: albumin level; Cr: creatinine; EOS%: eosinophil percentage; LDH: lactate dehydrogenase; LYM%: lymphocyte percentage; NEUT%: neutrophil percentage; PT: prothrombin time; SHAP: SHapley Additive exPlanations; T-Bil: total bilirubin.

Based on the SHAP algorithm, the feature ranking interpretation of the XGBoost model (Figure 7) shows that LDH, PT, Cr, LYM%, NEUT%, EOS%, T-Bil, and ALB were the characteristics of the XGBoost model with the greatest impact in predicting outcomes. Overall, the characteristics of LDH, PT, Cr, T-Bil, and NEUT% correlated positively with the outcomes and are risk factors; meanwhile, LYM%, EOS%, and ALB correlated negatively with the outcomes and are protective factors.

**Figure 8:**
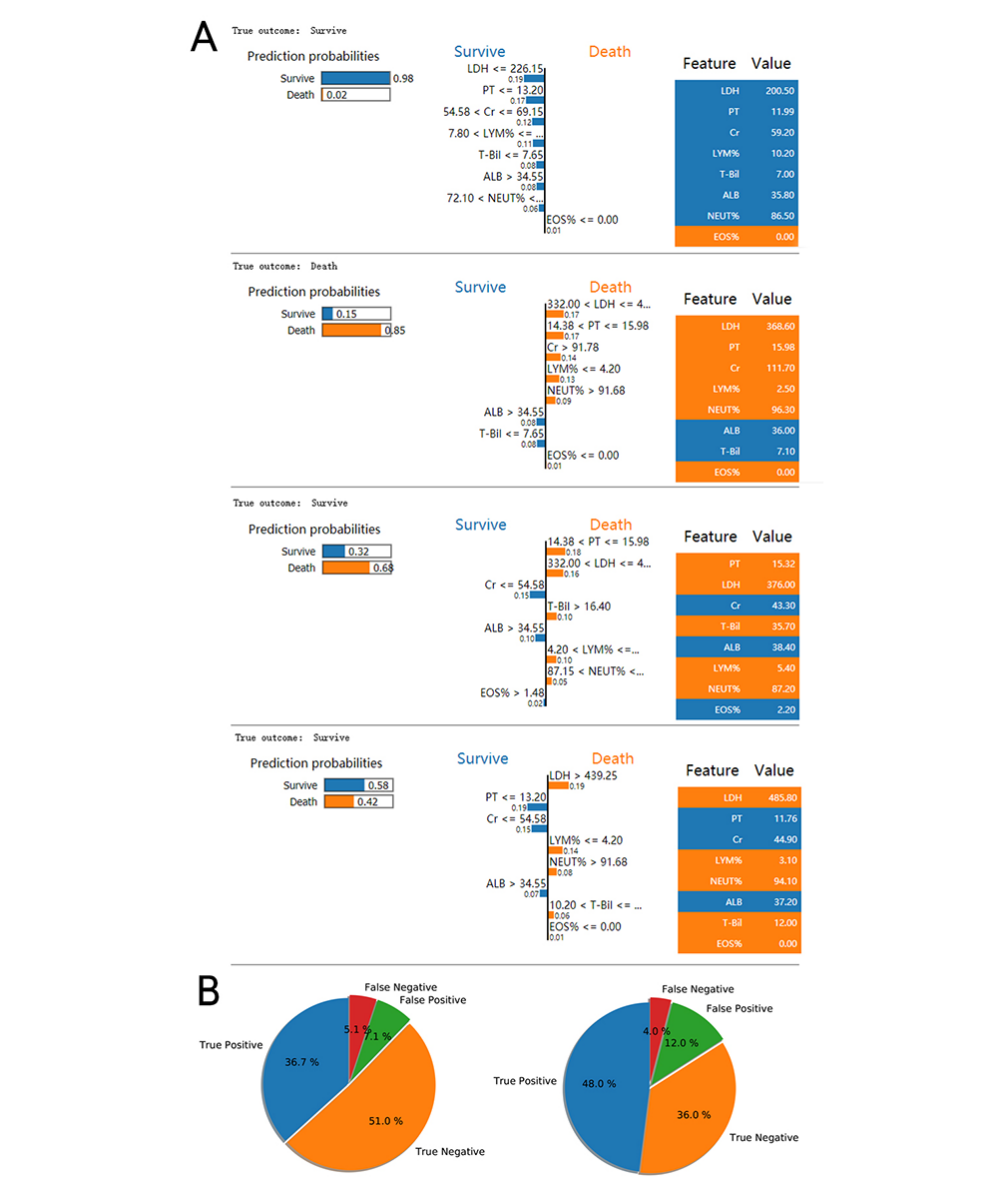
Interpretation of sample prediction results by randomly drawing samples to make model predictions and observing the model through the Local Interpretable Model-Agnostic Explanations (LIME) algorithm. (A) The four different prediction behaviors of the model (true negative, true positive, false negative, and false positive); (B) the ratios of the four prediction behaviors of the model on the training set and the test set. A:LB: albumin level; CR: creatinine: EOS%: eosinophil percentage; LDH: lactate dehydrogenase; LYM%: lymphocyte percentage; NEUT%: neutrophil percentage; PT: prothrombin time; T-Bil: total bilirubin.

Interpretation of sample prediction results requires random drawing of samples to make model predictions and observe the model through the LIME algorithm. The four prediction scenarios are shown in Figure 8A. In addition, the prediction results of the XGBoost model on all samples on the training set and the test set were counted, and the distribution of the four cases of the aggregated prediction results was visualized. Figure 8B shows that the XGBoost model has inappropriate prediction behavior in that its judgment of the false positive prediction results found through LIME is inaccurate; however, this situation is very rare, which indicates that the performance of the XGBoost prediction model is stable and reliable and that the interpretation of random sample prediction is basically reasonable. This is sufficient to confirm the practicability of the XGBoost model and will help increase physicians’ trust in the prediction model and help them make good auxiliary decisions.

**Figure 9:**
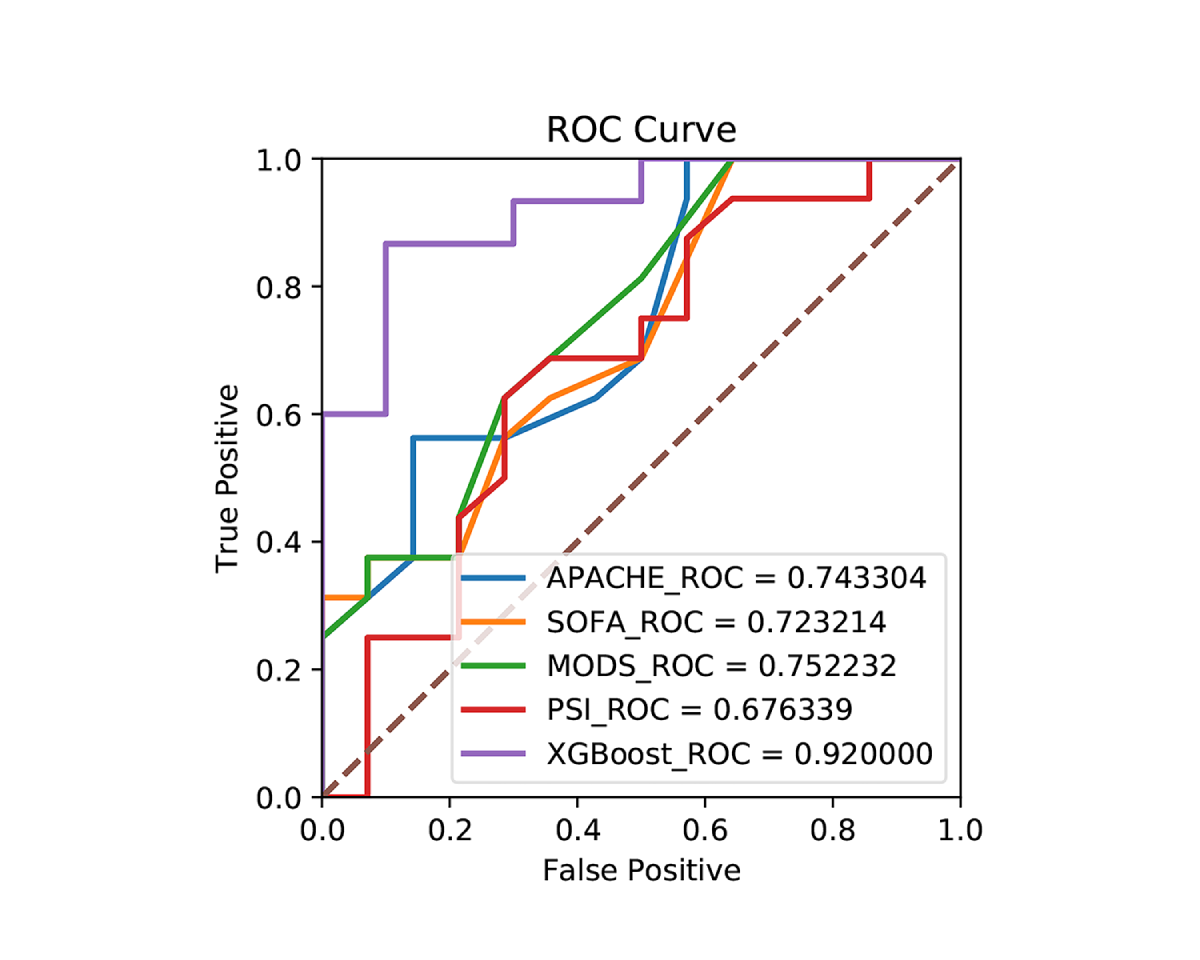
Comparison between the risk prediction model of the present study and the ROC curves of various critical scores. APACHE: Acute Physiologic Assessment and Chronic Health Evaluation; MODS: Multiple Organ Disfunction Score; PSI: Pneumonia Severity Index; ROC: receiver operating characteristic; SOFA: Sequential Organ Failure Assessment; XGBoost: eXtreme Gradient Boosting.

Additionally, comparison of the results of our machine learning model and the Acute Physiologic Assessment and Chronic Health Evaluation II (APACHE II), Sequential Organ Failure Assessment (SOFA), Multiple Organ Disfunction Score (MODS), and Pneumonia Severity Index (PSI) scores indicated that the AUC of the XGBoost model was higher than those of the other four scores (Figure 9).

## Discussion

### Principal Findings

The results of the present study show that the XGBoost method is a more reliable and more accurate method for predicting outcomes for critically ill patients with COVID-19 in the ICU than conventional logistic regression and scoring. Especially, the eigenvalues were reduced using the XGBoost model from 100 parameters to 8. Correlation analysis and characteristic analysis showed that the LDH, PT, Cr, T-Bil, LYM%, ALB, NEUT%, and EOS% indicators had strong correlations with the prognosis of severe and critical patients with COVID-19 in the ICU. Physicians should be wary of poor prognosis when encountering such patients. After full verification by SHAP, LIME, etc, the model was found to be accurate and stable. A web-based calculator based on the risk model is available on the internet [32].

According to the XGBoost algorithm model used in our study, LDH, PT, Cr, T-Bil, and NEUT% correlated positively with patients’ outcomes, indicating that these values are risk factors; meanwhile, LYM%, ALB, and EOS% correlated negatively with patients’ outcomes, indicating that these values are protective factors. In addition to identifying risk and protective factors, the results suggested that the XGBoost algorithm model achieves a good prediction effect, with an AUC of 0.92, sensitivity of 0.8, and specificity of 0.9. Based on the same data, logistic regression analysis showed an AUC of 0.84, with a sensitivity of 0.7 and specificity of 0.83. These results indicate that the predictive effect of machine learning is more accurate and sensitive than that of regression analysis. In contrast to our study, other researchers tended to apply Cox regression and logistic regression to analyze risk factors. Wang et al [19] found that the risk factors for in-hospital mortality from COVID-19 were lymphopenia and LDH, as analyzed by multivariable Cox proportional hazard regression models. Chen and colleagues [20] studied 1859 patients with conﬁrmed COVID-19 from seven centers in Wuhan, China, of whom 1651 recovered and 208 died. Multivariable Cox regression analyses indicated that increased hazards of in-hospital death were associated with one indicator, log 10 serum creatinine (sCr) per μ mol/L increase [33]. In another study that analyzed 167 confirmed patients with severe COVID-19, the LDH concentration was higher and the albumin concentration was lower in these patients, with significant differences [34]. Recently, Liang et al [22] used Least Absolute Shrinkage and Selection Operator (LASSO) and logistic regression to construct a predictive risk score (COVID-GRAM), in which the AUC in the development cohort was 0.88 (95% CI 0.85-0.91) and the AUC in the validation cohort was 0.88 (95% CI 0.84-0.93) for predicting patients’ risk of developing critical illness. The results in that study coincide with our results to some extent, and the machine learning algorithm can identify potential indicators better than the conventional algorithm. Finally, Abdulaal et al [20] used a neural network model to predict the prognosis of patients with COVID-19, with an AUC of 90.12%. This study only used eight indicators and achieved good predictive value; thus, it is more convenient and efficient.

Elevated LDH was identified as a significant risk predictive marker of COVID-19. Li et al [23] revealed that relatively high levels of LDH play a crucial role in predicting mortality of patients with COVID-19 when using an interpretable mortality prediction model. LDH is an enzyme that is involved in energy production through the conversion of lactate to pyruvate; it is present in almost all body cell types (n=156), with the highest levels in the heart, liver, lungs, muscles, kidneys, and blood cells. LDH is released from cells upon damage to their cytoplasmic membrane, and it is not only a metabolic marker but also an immune surveillance prognostic biomarker [35]. LDH increases the production of lactate, which leads to enhancement of immune-suppressive cells and inhibition of cytolytic cells. These changes weaken the immune response mounted against viral infection, which results in more severe disease in patients with COVID-19 who have elevated LDH [36]. PT is another typical indicator associated with patient prognosis. Through pathological examination of patients who died of COVID-19, researchers found that the virus can lead to disorders of the coagulation system, resulting in a hypercoagulable state and microthrombosis [37]. Moreover, viral infections may induce even more severe complications, such as acute respiratory distress syndrome and multi-organ dysfunction syndrome, which are two conditions frequently associated with hypercoagulation and disseminated intravascular coagulation [38]. These processes and conditions help to explain why PT was prolonged in patients with severe and critical COVID-19. Approximately 14.4% of patients with COVID-19 have elevated sCr levels, and kidney disease has been associated with in-hospital death of patients with COVID-19 [39]. SARS-CoV2 has been suggested to modulate the renin-angiotensin-aldosterone system (RAAS). Evidence of activation of the RAAS in patients with COVID-19 who have acute kidney injury, leading to increased sCr, has been reported [40]. Several studies have also reported that liver damage occurred in severe cases of COVID-19 infection at rates ranging from 58% to 78% [41,42]. COVID-19 uses angiotensin converting enzyme 2 (ACE2) as the binding site to enter host cells in the lungs, kidneys, and heart. A previous study [43] showed that both liver and bile duct cells express ACE2; this may result in elevated T-Bil levels, accompanied by slightly decreased ALB levels. Hematologic and immunologic impairment showed significantly different profiles between survival and mortality of patients with COVID-19 with different disease severities. The results of our study suggest that increased NEUT% and decreased LYM% are risk factors for patient prognosis. Interestingly, a decrease in EOS% was also a risk factor, and we were surprised to find that the results of two studies [44,45] were consistent with our results. In those studies, it was found that eosinophils decreased at the early stage and were associated with disease severity and clinical outcomes. Impaired immune cell function leads to low lymphocyte levels and immune system dysfunction, causing patients with severe COVID-19 to be more sensitive to bacterial infection [46]. The decline in eosinophils may be due to the patients’ response to the stress of acute SARS-CoV-2 infection. However, whether COVID-19 has a direct effect on eosinophils remains unknown. In one study, it was found that the *Clostridioides difficile* transferase toxin induces pathogenic host inflammation via a toll-like receptor 2–dependent pathway, resulting in suppression of the protective host eosinophilic response [47]. Additionally, eosinophils can be reduced after an innate immune challenge [48].

### Limitations

This study has several limitations. First, data from only one center were used, and the sample size was small, which may indicate bias. However, Vulcan Hill Hospital is a large medical center that focused on the treatment of COVID-19. The patients are representative of all patients with COVID-19, providing a reliable basis for the treatment of critical patients. Second, the treatment of patients in the ICU is not necessarily the initial treatment because the patients were transferred from different hospitals and different medical treatment units; this may have affected the baseline characteristics of the patients. In the future, based on what we learned in this study, we will attempt to correct defects of the model and the machine learning approach. Also, we will collect more data to conduct external tests on the prediction model and further improve the generalized prediction ability of the model for multicenter data.

### Conclusions

Machine learning has a good predictive effect on the mortality of critically ill patients with COVID-19 in the ICU. The XGBoost model has higher diagnostic performance than conventional statistical methods and can be used to select and simplify the core indicators for mortality prediction, such as LDH, PT, Cr, T-Bil, LYM%, ALB, and the white blood cell parameters NEUT% and BASO%. Machine learning may be a valuable prognostic indicator for early warning of critically ill patients; this warning plays a significant role in the allocation of medical resources, triage of patients, formulation of treatment decisions, and evaluation of progressive COVID-19.

## Appendix

Multimedia Appendix 1 Summary table of the number and proportion of missing values.

Multimedia Appendix 2 Mathematical algorithm.

Multimedia Appendix 3 Comparative analysis of all potential risk factors in the study cohort.

Multimedia Appendix 4 Correlation coefficient matrix heat maps.

Multimedia Appendix 5 Load matrix diagram showing the correlations between the characteristics and the main factors.

## Abbreviations

ACE2: angiotensin converting enzyme 2
AdaBoost: adaptive boosting
ALB: albumin level
APACHE II: Acute Physiologic Assessment and Chronic Health Evaluation II
AUC: area under the receiver operating characteristic curve
BASO%: basophil percentage
Cr: creatinine
EOS%: eosinophil percentage
GBDT: gradient boosting decision tree
ICU: intensive care unit
LASSO: Least Absolute Shrinkage and Selection Operator
LDH: lactate dehydrogenase
LYM%: lymphocyte percentage
MODS: Multiple Organ Disfunction Score
NEUT%: neutrophil percentage
NLR: negative likelihood ratio
NPV: negative predictive value
PLR: positive likelihood ratio
PPV: positive predictive value
PROBAST: Prediction model Risk Of Bias ASsessment Tool
PSI: Pneumonia Severity Index
PT: prothrombin time
RAAS: renin-angiotensin-aldosterone system
sCr: serum creatinine
SHAP: SHapley Additive exPlanations
SOFA: Sequential Organ Failure Assessment
T-Bil: total bilirubin
TRIPOD: Transparent Reporting of a multivariable prediction model for Individual Prognosis Or Diagnosis
XGBoost: eXtreme Gradient Boosting
