# Supplementary material for: Prognostic Assessment of COVID-19 in the Intensive Care Unit by Machine Learning Methods: Model Development and Validation
Source: J Med Internet Res. 2020 Nov 11;22(11):e23128. doi: 10.2196/23128 (PMC7661105; doi:10.2196/23128)
Supplement: Multimedia Appendix 1 [file jmir_v22i11e23128_app1.doc]

Appendix 1 Summary table of the number and proportion of missing values

| **Variable** | **Nnm of Missing** | **Percent of Missing(%)** |
| --- | --- | --- |

| β2-MG | 123 | 100.0 |
| --- | --- | --- |
| P-ALB | 123 | 100.0 |
| MB | 122 | 99.2 |
| h-TnI | 116 | 94.3 |
| Nucleic acid results within three days after admission | 39 | 31.7 |
| HR | 33 | 26.8 |
| PCT | 16 | 13.0 |
| PTA% | 15 | 12.2 |
| BNP | 14 | 11.4 |
| CK-MB | 13 | 10.6 |
| D-Dimer | 13 | 10.6 |
| CK | 12 | 9.8 |
| AST | 12 | 9.8 |
| FIB | 12 | 9.8 |
| LDH | 12 | 9.8 |
| α-HBDH | 12 | 9.8 |
| total CO2 | 12 | 9.8 |
| BUN | 11 | 8.9 |
| Cr | 11 | 8.9 |
| GLU | 11 | 8.9 |
| APTT | 11 | 8.9 |
| PT | 11 | 8.9 |
| TT | 11 | 8.9 |
| INR | 11 | 8.9 |
| UA | 11 | 8.9 |
| cys-c | 11 | 8.9 |
| γ-GT | 8 | 6.5 |
| ALP | 8 | 6.5 |
| Mg | 8 | 6.5 |
| K | 8 | 6.5 |
| N | 8 | 6.5 |
| P | 8 | 6.5 |
| Ca | 8 | 6.5 |
| Cl | 8 | 6.5 |
| A/G ratio | 8 | 6.5 |
| Total bile acids | 8 | 6.5 |
| I-Bil | 8 | 6.5 |
| D-Bil | 8 | 6.5 |
| T-Bil | 8 | 6.5 |
| GLB | 8 | 6.5 |
| ALB | 8 | 6.5 |
| TP | 8 | 6.5 |
| ALT | 8 | 6.5 |
| UO | 4 | 3.3 |
| CRP | 4 | 3.3 |
| ICU-stay time | 3 | 2.4 |
| HCT | 2 | 1.6 |
| HGB | 2 | 1.6 |
| PLT | 2 | 1.6 |
| BASO | 2 | 1.6 |
| EOS | 2 | 1.6 |
| MONO | 2 | 1.6 |
| LYM | 2 | 1.6 |
| NEUT | 2 | 1.6 |
| BASO% | 2 | 1.6 |
| EOS% | 2 | 1.6 |
| MONO% | 2 | 1.6 |
| LYM% | 2 | 1.6 |
| NEUT% | 2 | 1.6 |
| WBC | 2 | 1.6 |
| RBC | 2 | 1.6 |
