# Supplementary material for: Prognostic Assessment of COVID-19 in the Intensive Care Unit by Machine Learning Methods: Model Development and Validation
Source: J Med Internet Res. 2020 Nov 11;22(11):e23128. doi: 10.2196/23128 (PMC7661105; doi:10.2196/23128)
Supplement: Multimedia Appendix 2 [file jmir_v22i11e23128_app2.docx]

Appendix 2 Mathematical algorithm

| algorithm | Parameters |
| --- | --- |
| XGBoost | XGBClassifier(base_score=0.5, booster='gbtree', colsample_bylevel=1, colsample_bynode=1, colsample_bytree=0.8, gamma=0, learning_rate=0.1, max_delta_step=0, max_depth=2, min_child_weight=4.5, missing=None, n_estimators=260, n_jobs=1, nthread=None, objective='binary:logistic', random_state=0,  reg_alpha=0.1, reg_lambda=1, scale_pos_weight=1, seed=None, silent=None, subsample=0.7, verbosity=1) |
| LogisticRegression | LogisticRegression(C=1.0, class_weight=None, dual=False, fit_intercept=True, intercept_scaling=1, l1_ratio=None, max_iter=100, multi_class='warn', n_jobs=None, penalty='l2', random_state=None, solver='warn', tol=0.0001, verbose=0, warm_start=False) |
| GBDT | GradientBoostingClassifier(criterion='friedman_mse', init=None, learning_rate=0.1, loss='deviance', max_depth=3, max_features=8, max_leaf_nodes=None, min_impurity_decrease=0.0, min_impurity_split=None, min_samples_leaf=33, min_samples_split=20, min_weight_fraction_leaf=0.0, n_estimators=28, n_iter_no_change=None, presort='auto', random_state=0, subsample=0.9, tol=0.0001, validation_fraction=0.1, verbose=0, warm_start=False) |
| AdaBoost | AdaBoostClassifier(algorithm='SAMME.R', base_estimator=DecisionTreeClassifier(class_weight=None, criterion='gini', max_depth=3, max_features=1, max_leaf_nodes=None, min_impurity_decrease=0.0, min_impurity_split=None,  min_samples_leaf=20, min_samples_split=15, min_weight_fraction_leaf=0.0, presort=False, random_state=0, splitter='best'),  learning_rate=0.1, n_estimators=14, random_state=0) |
| CatBoost | CatBoostClassifier('depth': 3, 'iterations': 50, 'learning_rate': 0.05, 'l2_leaf_reg': 50,  'scale_pos_weight': 1, 'thread_count': 4, 'random_seed': 0) |
