# Supplementary material for: Prognostic Assessment of COVID-19 in the Intensive Care Unit by Machine Learning Methods: Model Development and Validation
Source: J Med Internet Res. 2020 Nov 11;22(11):e23128. doi: 10.2196/23128 (PMC7661105; doi:10.2196/23128)
Supplement: Multimedia Appendix 3 [file jmir_v22i11e23128_app3.doc]

| **Appendix 3 Comparative analysis of all potential risk factors in the study cohort**  a Related features of patient baseline information | | | | |
| --- | --- | --- | --- | --- |
|  | | **Death** | **Survive** | **P-value** |
| **Variables** | | 58 | 65 | - |
| Gender, n (%) | Female | 17（29.31%） | 27（41.54%） | .221^a^ |
|  | Male | 41（70.69%） | 38（58.46%） |  |
| Direct ICU admission, n (%) | No | 45（77.59%） | 39（60.0%） | .058^a^ |
|  | Yes | 13（22.41%） | 26（40.0%） |  |
| Infection types , n (%) | Non-close contact | 27（46.55%） | 30（46.15%） | .891^a^ |
|  | Close contact | 31（53.45%） | 35（53.85%） |  |
| Nucleic acid positive on admission, n (%) | No | (11, 18.97) | (22, 33.85) | .098^a^ |
|  | Yes | (47, 81.03) | (43, 66.15) |  |
| ICU-stay time, (median, quantile (0.25,0.75)) | | (6.0, (3.0, 13.0)) | (8.0, (3.0, 13.0)) | 0.252 ^b^ |
| Age, (mean±sd） | | (71.276, 9.256) | (68.431, 9.256) | 0.157 ^b^ |
| Hospital-stay time, (median, quantile (0.25,0.75)) | | (12.0, (6.0, 21.0)) | (25.0, (6.0, 21.0)) | 0 ^b^ |
| Severe illness, n (%) | No | (0.0, 0.0) | (5.0, 7.69) | .098^a^ |
|  | Yes | (58.0, 100.0) | (60.0, 92.31) |  |
| Critical illness, n (%) | No | (5, 8.62) | (33, 50.77) | .0^a^ |
|  | Yes | (53, 91.38) | (32, 49.23) |  |
|  | |  |  |  |

| b Related features of clinical diagnosis | | | | |
| --- | --- | --- | --- | --- |
|  | | **Death** | **Survive** | **P-value** |
| **Variables** | | 58 | 65 | - |
| Hypertension, n (%) | No | 30（51.72%） | 29（44.62%） | .544^a^ |
|  | Yes | 28（48.28%） | 36（55.38%） |  |
| Diabetes, n (%) | No | (43, 74.14) | (56, 86.15) | .0147^a^ |
|  | Yes | (15, 25.86) | (9, 13.85) |  |
| Liver disease, n (%) | No | (53, 91.38) | (55, 84.62) | .385^a^ |
|  | Yes | (5, 8.62) | (10, 15.38) |  |
| Chronic heart disease, n (%) | No | (54, 93.1) | (62, 95.38) | .877^a^ |
|  | Yes | (4, 6.9) | (3, 4.62) |  |
| Malignant tumor, n (%) | No | (57, 98.28) | (64, 98.46) | .527^a^ |
|  | Yes | (1, 1.72) | (1, 1.54) |  |
| Structural pulmonary disease, n (%) | No | (55, 94.83) | (54, 83.08) | .078^a^ |
|  | Yes | (3, 5.17) | (11, 16.92) |  |
| Immunosuppressed population, n (%) | No | (58.0, 100.0) | (64.0, 98.46) | .954^a^ |
|  | Yes | (0.0, 0.0) | (1.0, 1.54) |  |
|  | |  |  |  |

| c Related characteristics of vital signs and nursing | | | | |
| --- | --- | --- | --- | --- |
|  | | **Death** | **Survive** | **P-value** |
| **Variables** | | 58 | 65 | - |
| ventilator, n (%) | No | 5（8.62%） | 35（53.85%） | .0^a^ |
|  | Yes | 53（91.38%） | 30（46.15%） |  |
| UO, (median, quantile (0.25,0.75)) | | 800.0, (400.0, 1100.0) | 800.0, (400.0, 1100.0) | 0.198 ^b^ |
| SBp, (mean±sd） | | (133.483, 23.532) | (131.938, 23.532) | 0.682 ^b^ |
| Pulse, (median, quantile (0.25,0.75)) | | (100.0, (85.25, 110.0)) | (84.0, (85.25, 110.0)) | 0 ^b^ |
| RR, (median, quantile (0.25,0.75)) | | (26.0, (20.0, 30.0)) | (21.0, (20.0, 30.0)) | 0.001 ^b^ |
| DBp, (median, quantile (0.25,0.75)) | | (78.0, (69.25, 88.0)) | (75.0, (69.25, 88.0)) | 0.257 ^b^ |
| SpO2, (median, quantile (0.25,0.75)) | | (94.0, (88.0, 97.0)) | (98.0, (88.0, 97.0)) | 0 ^b^ |
| T, (median, quantile (0.25,0.75)) | | (36.6, (36.3, 37.0)) | (36.5, (36.3, 37.0)) | 0.175 ^b^ |
| HR, (median, quantile (0.25,0.75)) | | (97.5, (84.0, 108.75)) | (80.0, (84.0, 108.75)) | 0 ^b^ |
|  | |  |  |  |

| d Related features of treatment and drug application | | | | |
| --- | --- | --- | --- | --- |
|  | | **Death** | **Survive** | **P-value** |
| **Variables** | | 58 | 65 | - |
| ECMO, n (%) | No | 53（91.38%） | 65（100.0%） | .05^a^ |
|  | Yes | 5（8.62%） | 0（0.0%） |  |
| Recovery plasma, n (%) | No | 53（91.38%） | 57（87.69%） | .711^a^ |
|  | Yes | 5（8.62%） | 8（12.31%） |  |
| Hemodialysis filtration, n (%) | No | (43, 74.14) | (64, 98.46) | .0^a^ |
|  | Yes | (15, 25.86) | (1, 1.54) |  |
| Antiviral therapy, n (%) | No | (30, 51.72) | (36, 55.38) | .822^a^ |
|  | Yes | (28, 48.28) | (29, 44.62) |  |
| Vasopressors, n (%) | No | (7, 12.07) | (61, 93.85) | .0^a^ |
|  | Yes | (51, 87.93) | (4, 6.15) |  |
| Glucocorticoid, n (%) | No | (44, 75.86) | (41, 63.08) | .181^a^ |
|  | Yes | (14, 24.14) | (24, 36.92) |  |
| Enzyme inhibitor, n (%) | No | (37, 63.79) | (37, 56.92) | .554^a^ |
|  | Yes | (21, 36.21) | (28, 43.08) |  |
| Carbapenem, n (%) | No | (31, 53.45) | (55, 84.62) | .0^a^ |
|  | Yes | (27, 46.55) | (10, 15.38) |  |
| General antibiotics, n (%) | No | (56, 96.55) | (59, 90.77) | .351^a^ |
|  | Yes | (2, 3.45) | (6, 9.23) |  |
| Antifungal therapy, n (%) | No | (46, 79.31) | (59, 90.77) | .124^a^ |
|  | Yes | (12, 20.69) | (6, 9.23) |  |
| Antibiotic resistant bacteria, n (%) | No | (51.0, 87.93) | (65.0, 100.0) | .013^a^ |
|  | Yes | (7.0, 12.07) | (0.0, 0.0) |  |
| Gram positive cocci , n (%) | No | (37, 63.79) | (59, 90.77) | .001^a^ |
|  | Yes | (21, 36.21) | (6, 9.23) |  |
| Anticoagulation, n (%) | No | (40, 68.97) | (39, 60.0) | .397^a^ |
|  | Yes | (18, 31.03) | (26, 40.0) |  |
| Quinolones, n (%) | No | (36, 62.07) | (40, 61.54) | .9^a^ |
|  | Yes | (22, 37.93) | (25, 38.46) |  |
| Chloroquine, n (%) | No | (57, 98.28) | (62, 95.38) | .694^a^ |
|  | Yes | (1, 1.72) | (3, 4.62) |  |
| Immunoglobulin, n (%) | No | (37, 63.79) | (50, 76.92) | .162^a^ |
|  | Yes | (21, 36.21) | (15, 23.08) |  |
| Immunomodulator, n (%) | No | (56, 96.55) | (60, 92.31) | .0532^a^ |
|  | Yes | (2, 3.45) | (5, 7.69) |  |
|  | |  |  |  |

| e Related characteristics of laboratory test | | | |
| --- | --- | --- | --- |
|  | **Death** | **Survive** | **P-value** |
| **Variables** | 58 | 65 | - |
| INR, (median, quantile (0.25,0.75)) | (1.32, (1.223, 1.462)) | (1.15, (1.223, 1.462)) | 0 ^b^ |
| Cr, (median, quantile (0.25,0.75)) | (79.0, (62.825, 123.325)) | (60.7, (62.825, 123.325)) | 0 ^b^ |
| BASO, (median, quantile (0.25,0.75)) | (0.0, (0.0, 0.01)) | (0.01, (0.0, 0.01)) | 0.002 ^b^ |
| TP, (mean±SD) | (58.826, 6.751) | (61.734, 6.751) | 0.018 ^b^ |
| RBC, (mean±SD) | (3.808, 0.816) | (3.751, 0.816) | 0.666 ^b^ |
| CK, (median, quantile (0.25,0.75)) | (89.1, (52.675, 185.9)) | (39.5, (52.675, 185.9)) | 0 ^b^ |
| GLU, (median, quantile (0.25,0.75)) | (8.75, (6.402, 13.685)) | (6.12, (6.402, 13.685)) | 0 ^b^ |
| Cl, (median, quantile (0.25,0.75)) | (104.85, (100.85, 110.1)) | (104.1, (100.85, 110.1)) | 0.069 ^b^ |
| P, (median, quantile (0.25,0.75)) | (0.895, (0.718, 1.138)) | (0.88, (0.718, 1.138)) | 0.419 ^b^ |
| N, (median, quantile (0.25,0.75)) | (142.9, (137.825, 146.175)) | (140.2, (137.825, 146.175)) | 0.033 ^b^ |
| cys-c, (median, quantile (0.25,0.75)) | (1.35, (1.042, 1.642)) | (1.1, (1.042, 1.642)) | 0.003 ^b^ |
| WBC, (median, quantile (0.25,0.75)) | (11.55, (7.375, 16.35)) | (7.7, (7.375, 16.35)) | 0 ^b^ |
| ALT, (median, quantile (0.25,0.75)) | (41.25, (24.425, 67.7)) | (24.1, (24.425, 67.7)) | 0.006 ^b^ |
| APTT, (median, quantile (0.25,0.75)) | (29.61, (26.47, 34.228)) | (28.05, (26.47, 34.228)) | 0.043 ^b^ |
| HGB, (median, quantile (0.25,0.75)) | (116.69, 24.351) | (114.446, 24.351) | 0.57 ^b^ |
| HCT, (median, quantile (0.25,0.75)) | (35.25, (31.075, 40.225)) | (34.6, (31.075, 40.225)) | 0.175 ^b^ |
| T-Bil, (median, quantile (0.25,0.75)) | (14.75, (10.1, 21.375)) | (8.6, (10.1, 21.375)) | 0 ^b^ |
| GLB, (median, quantile (0.25,0.75)) | (27.85, (24.675, 30.15)) | (27.6, (24.675, 30.15)) | 0.363 ^b^ |
| ALB, (mean±sd） | (30.679, 4.43) | (33.611, 4.43) | 0 ^b^ |
| γ-GT, (median, quantile (0.25,0.75)) | (52.15, (32.65, 102.975)) | (45.9, (32.65, 102.975)) | 0.051 ^b^ |
| NEUT%, (median, quantile (0.25,0.75)) | (91.3, (87.5, 93.85)) | (80.7, (87.5, 93.85)) | 0 ^b^ |
| MONO, (median, quantile (0.25,0.75)) | (0.4, (0.212, 0.588)) | (0.41, (0.212, 0.588)) | 0.478 ^b^ |
| D-Bil, (median, quantile (0.25,0.75)) | (7.9, (4.725, 12.35)) | (3.5, (4.725, 12.35)) | 0 ^b^ |
| A/G ratio, (median, quantile (0.25,0.75)) | (1.095, (0.96, 1.22)) | (1.2, (0.96, 1.22)) | 0.002 ^b^ |
| Ca, (median, quantile (0.25,0.75)) | (1.96, (1.87, 2.03)) | (2.1, (1.87, 2.03)) | 0 ^b^ |
| ALP, (median, quantile (0.25,0.75)) | (101.6, (87.075, 135.6)) | (77.8, (87.075, 135.6)) | 0 ^b^ |
| PLT, (median, quantile (0.25,0.75)) | (166.0, (79.25, 226.75)) | (224.0, (79.25, 226.75)) | 0 ^b^ |
| PCT, (median, quantile (0.25,0.75)) | (0.45, (0.202, 1.228)) | (0.09, (0.202, 1.228)) | 0 ^b^ |
| LYM%, (median, quantile (0.25,0.75)) | (4.4, (2.9, 7.875)) | (11.7, (2.9, 7.875)) | 0 ^b^ |
| D-Dimer, (median, quantile (0.25,0.75)) | (5.445, (2.995, 7.92)) | (1.77, (2.995, 7.92)) | 0 ^b^ |
| α-HBDH, (median, quantile (0.25,0.75)) | (367.6, (292.35, 556.275)) | (213.6, (292.35, 556.275)) | 0 ^b^ |
| Total CO2, (mean±sd） | (22.777, 5.583) | (25.237, 5.583) | 0.01 ^b^ |
| UA, (median, quantile (0.25,0.75)) | (230.0, (167.75, 390.0)) | (209.0, (167.75, 390.0)) | 0.064 ^b^ |
| Total bile acids, (median, quantile (0.25,0.75)) | (3.15, (2.4, 5.9)) | (3.6, (2.4, 5.9)) | 0.421 ^b^ |
| BUN, (median, quantile (0.25,0.75)) | (9.6, (6.212, 15.132)) | (6.09, (6.212, 15.132)) | 0 ^b^ |
| I-Bil, (median, quantile (0.25,0.75)) | (7.14, (4.42, 10.135)) | (5.38, (4.42, 10.135)) | 0.025 ^b^ |
| LDH, (median, quantile (0.25,0.75)) | (437.3, (336.9, 636.45)) | (251.0, (336.9, 636.45)) | 0 ^b^ |
| CRP, (median, quantile (0.25,0.75)) | (92.27, (31.49, 158.68)) | (13.87, (31.49, 158.68)) | 0 ^b^ |
| K, (mean±sd） | (4.42, 0.761) | (4.248, 0.761) | 0.171 ^b^ |
| PT, (median, quantile (0.25,0.75)) | (15.815, (14.645, 17.542)) | (13.82, (14.645, 17.542)) | 0 ^b^ |
| EOS, (median, quantile (0.25,0.75)) | (0.01, (0.0, 0.06)) | (0.07, (0.0, 0.06)) | 0.006 ^b^ |
| BASO%, (median, quantile (0.25,0.75)) | (0.0, (0.0, 0.1)) | (0.2, (0.0, 0.1)) | 0 ^b^ |
| NEUT, (median, quantile (0.25,0.75)) | (10.215, (6.115, 15.06)) | (6.03, (6.115, 15.06)) | 0 ^b^ |
| CK-MB, (median, quantile (0.25,0.75)) | (19.1, (13.325, 31.65)) | (11.6, (13.325, 31.65)) | 0 ^b^ |
| PTA%, (median, quantile (0.25,0.75)) | (82.6, (76.775, 87.125)) | (90.7, (76.775, 87.125)) | 0 ^b^ |
| BNP, (median, quantile (0.25,0.75)) | (106.81, (42.41, 401.935)) | (46.57, (42.41, 401.935)) | 0 ^b^ |
| EOS%, (median, quantile (0.25,0.75)) | (0.1, (0.0, 0.6)) | (0.9, (0.0, 0.6)) | 0 ^b^ |
| Mg, (median, quantile (0.25,0.75)) | (0.98, (0.883, 1.102)) | (0.89, (0.883, 1.102)) | 0 ^b^ |
| FIB, (mean±sd） | (2.948, 1.417) | (3.308, 1.417) | 0.072 ^b^ |
| AST, (median, quantile (0.25,0.75)) | (36.35, (23.45, 64.675)) | (25.4, (23.45, 64.675)) | 0.001 ^b^ |
| TT, (median, quantile (0.25,0.75)) | (17.32, (15.665, 19.555)) | (15.54, (15.665, 19.555)) | 0 ^b^ |
| MONO%, (median, quantile (0.25,0.75)) | (3.35, (2.1, 5.375)) | (5.5, (2.1, 5.375)) | 0 ^b^ |
| LYM, (median, quantile (0.25,0.75)) | (0.57, (0.352, 0.865)) | (0.84, (0.352, 0.865)) | 0 ^b^ |
|  |  |  |  |
