# Supplementary material for: Prognostic Assessment of COVID-19 in the Intensive Care Unit by Machine Learning Methods: Model Development and Validation
Source: J Med Internet Res. 2020 Nov 11;22(11):e23128. doi: 10.2196/23128 (PMC7661105; doi:10.2196/23128)

## Multimedia Appendix 4

Correlation coefficient matrix heat maps showing the Spearman correlations between factors and machine learning features and between factors and outcomes; the darker the color, the stronger the correlation. For ease of observation, the 45 continuous variables were divided into two graphs (A and B).


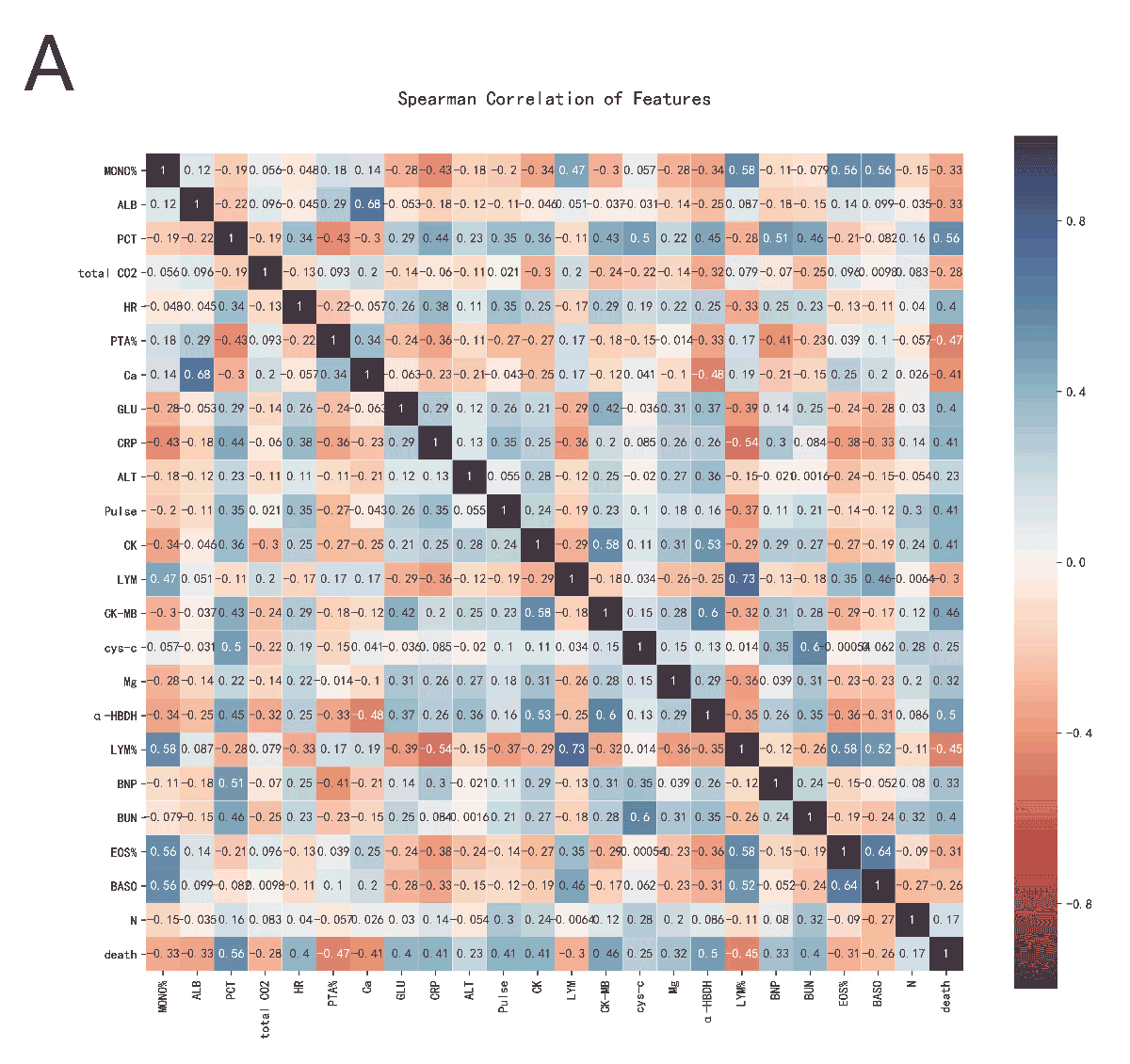


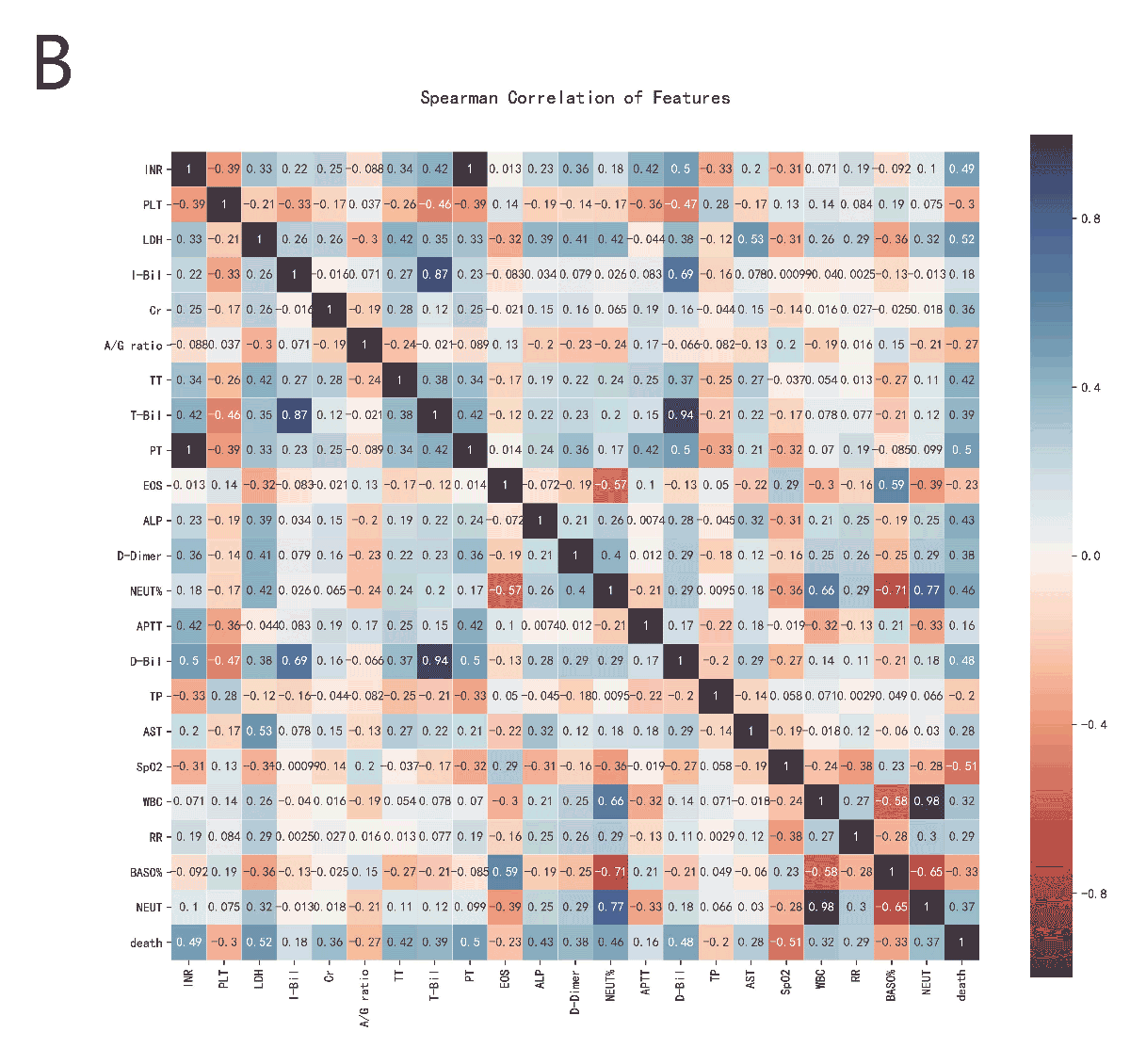

Supplement: Multimedia Appendix 4 [file jmir_v22i11e23128_app4.docx]
