# Supplementary figures and images for: Prognostic Assessment of COVID-19 in the Intensive Care Unit by Machine Learning Methods: Model Development and Validation
Source: J Med Internet Res. 2020 Nov 11;22(11):e23128. doi: 10.2196/23128 (PMC7661105; doi:10.2196/23128)

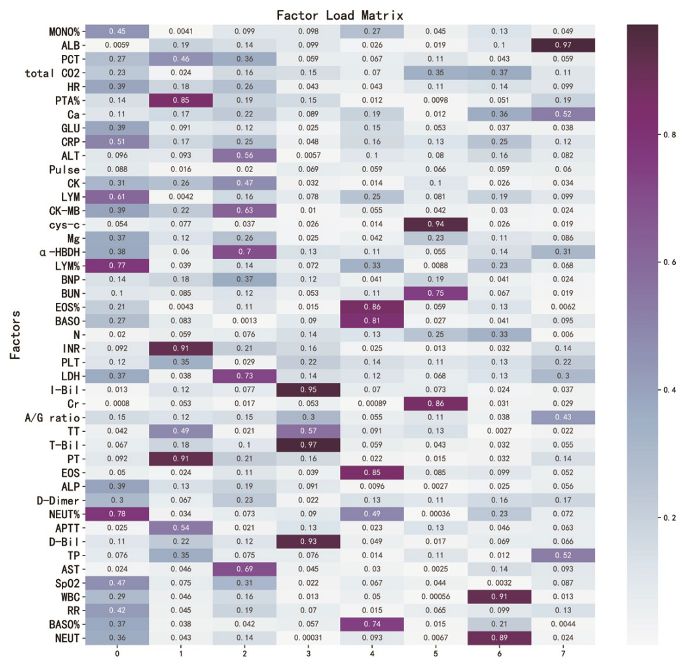

Supplement: Multimedia Appendix 5 [file jmir_v22i11e23128_app5.png]
